# Ultrafine particles in scanning sprays: a standardized examination of five powders used for dental reconstruction

**DOI:** 10.1186/s12995-020-00271-2

**Published:** 2020-06-29

**Authors:** E. Ochsmann, P. Brand, T. Kraus, S. Reich

**Affiliations:** 1 Institute for Occupational, Social and Environmental Medicine, Faculty of Medicine, RWTH University Hospital, Aachen, Germany; 2 Institute for Occupational Medicine, Prevention and Occupational Health Management, University of Lübeck, Lübeck, Germany; 3 Department of Prosthodontics, Implantology and Biomaterials, Faculty of Medicine, RWTH University Hospital, Aachen, Germany

**Keywords:** Nanoparticles, Dentistry, Risk assessment

## Abstract

**Background:**

Intraoral matting sprays for chairside systems can release fine or ultrafine particles or nanoparticles at dentists’ workplaces and cause work-related health problems by inhalation exposure. Until now, little is known about the magnitude of the ultrafine fraction, when using these scanning sprays. Hence, more information is needed for workplace risk assessments in dental practices.

**Methods:**

Five commonly used dental spray-powders were examined under standardized conditions. Ingredients were taken from the respective safety data sheet. Particle number-size distributions and total number concentrations were analyzed with a fast mobility particle sizer, and reported graphically as well as mean particle fractions smaller than 100 nm. Based on these measurements, risk assessments were conducted, and particle depositions in the lung were modelled.

**Results:**

The mean fraction of particles smaller than 100 nm varied between 9 and 93% depending on the matting agent and mode of application of the intraoral scanning spray. Propellants can represent a large fraction of these particles. Titanium dioxide, pigment-suspensions, talcum and others particles, which can pose relevant health risks, were listed as ingredients of scanning sprays in safety data sheets. Nevertheless, the deposited fraction of hazardous particles in the lung of employees in dental practices seems to be small (15%) during this dental procedure.

**Conclusions:**

Our results suggest that dentists’ personnel can be exposed to hazardous fine and ultrafine particles. Though extensive standardized measurements and systematic evaluation of safety data sheets were used for this study, they cannot sufficiently assess and categorize potential workplace-related health risks.

## Background

Fine and ultrafine particles, as well as manufactured nanoparticles are used or can be released in dentistry. In most cases, the term “nano-dentistry” refers to the properties of manufactured nanomaterials to maintain oral health care up to a high extent [1]. Nanomaterials can be found in pastes or can be set free during intraoral processing of a dental implant or because of the abrasion during in-period use [2]. But the resulting exposure of patients and personnel to these manufactured nanoparticles, are not in the focus of this study. Instead this study focuses on the use of matting sprays in dental reconstruction and on the accompanied involuntary inhalation exposure of dentists (and eventually patients) to fine and ultrafine particles.

In dental reconstruction, optical intraoral impression devices (**i**ntra**o**ral **s**canners, IOS) were introduced in the late 1980ies. One of the first was the camera of the Cerec 1 unit (**C**hairside **E**conomical **R**estoration of **E**sthetic **C**eramics [3];). Since the beginning of the twenty-first century, coinciding with the ongoing development of CAD/CAM technology (CAD: computer-aided design; CAM: computer-aided manufacturing), the number of commercially available intraoral scanning systems has been continually increasing [4–6]. Accompanying this technical development was the development of different scanning sprays for the respective chairside system. As dental tissues present many reflecting surfaces, some of these IOS systems need a 20–40 μm coating with a matting agent (usually applied by an intraoral spraying process) to reduce reflectivity [7, 8]. This process exposes patients, as well as dental personnel to spray mists. Although not initially classified as a nanotechnology-based products, the matting sprays in use can contain airborne ultrafine fractions [9], and therefore, the process of coating the teeths’ surfaces with a matting agent before taking the optical impression, represent a means of exposure to fine and ultrafine particles in dentists, dental personnel and patients [9].

The sprays, powders and applications used for the different IOS systems are released as medical products for optical impression taking. Until now, only few studies report particle (size) distributions of dental sprays, especially in the context of occupational health risk assessments [9]. Nevertheless, there are other studies regarding the application of ready-to-use sprays containing ultrafine fractions [10]. They find that, in general, the total inhalation exposure is larger for powder exposures than for spray exposures, whereas the ultrafine fraction is more dominant in spray applications compared to powders [10]. These studies also showed that a) sprays not claiming to contain manufactured nanomaterials, nevertheless produce inhalable fine and ultrafine fractions, and that b) the inhalation of ultrafine particles leads to deposition of these particles all over the respiratory tract, where they can permeate into and through mucosa and cause detrimental health effects, e. g. local and systemic inflammatory effects [11–13].

Nano- or ultrafine particles in general are said to aggravate pre-existing respiratory diseases [14], and lead to lung fibrosis [15, 16]. Particle sizes of less than 100 nm in diameter are of special interest, as a respiratory tract model of the International Commission on Radiological protection (ICPR) has shown that particles deposited in the tracheal bronchus and alveoli most likely are less than 100 nm in diameter [17–19]. Apart from that, non-degradable ultrafine particles could accumulate in the body and could cause unwanted effects in the lung and all other organs of the body. Another key issue, mostly resulting from animal experiments is a potentially carcinogenic effect of ultrafine particles, which can depends on underlying substances and is still under scrutiny [13, 20].

Because of these possible detrimental health effects, the use of intraoral scanning sprays should be considered in safety assessments of dental workplaces. Until now, only one study [9] directly examined exposures for dentists and patients when using one particular matting spray in an experimental setting. They found that the use of intraoral matting sprays in dentistry can lead to occupational as well as patient ultrafine inhalation exposure [9, 21, 22] and discuss that this might be associated with diseases in patient, dentist, and dental staff [9]. In their discussion they also demanded more information on particle distributions of other sprays. This study addresses this query and reports particle size distributions and particle number concentrations of five dental sprays, which are commonly used in dental practice. IT does so, to gain further insight into possible health hazards for health care personnel and dentists’ by fine and ultrafine particles. It also addresses the issue of workplace safety assessments with available information of material safety data sheets, and deposition modelling of fine and ultrafine particles in the respiratory tract.

## Methods

### Dental sprays

Altogether, five commonly used scanning sprays were analyzed (Table 1). The selection of sprays was determined by a dentist with experience with different chairside systems. Safety data sheets were used to gather available information on spray contents, scanning sprays’ ingredients, facets of exposure control and application modes. For this study, especially information of section 3 (composition/ information on ingredients) and section 8 (exposure control/ personal protection) of the respective safety data sheets were excerpted. Also, application procedures of the examined sprays are reported and compared.

**Table 1:** Safety data sheet information (sections 3 and 8) and mode of application of the used dental sprays

	Spray A	Spray B	Spray C	Spray D + spray D-propellant	Spray D-propellant	Spray E
Section in safety data sheet						
3. composition/ information on ingredients	heptafluoropropane ethanol n-pentane	ethanol heptafluoropropane naptha talcum citrus oil	pentane with non-hazardous additions	titanium dioxide zirconium dioxide, talcum	isobutene	titanium dioxide zirconium dioxide, zinc distearate
8. exposure control/ personal protection	ethanol n-pentane	ethanol talcum titanium dioxide silicium dioxide	pentane ethanol	titanium dioxide zirconium oxide talcum	isobutene	titanium dioxide
Mode of application						
	spray application	spray application	spray application	powder and propellant in separate containers, mixed when applied		application by an electrically driven pressured air source

### Risk assessment tool

For an approximated risk assessment, the information of the safety data sheets, along with probable exposure scenarios in dental practice were denoted in an online risk assessment tool, the “Stoffenmanager.Nano” (https://nano.stoffenmanager.com/Default.aspx [23];). The Stoffenmanager is a tool to qualitatively assess occupational health risks from inhalation exposure to manufactured nano objects (MNO). As most dental matting sprays do not explicitly refer to nanoparticles in their mixture, the use of “Stoffenmanager.Nano” shall only give a rough estimation of risks associated with ultrafine exposures.

In practice, spray content information are usually only available from safety data sheets. Information of transmission electron microscopy (TEM) examinations is scarce. That is why in this case all potentially hazardous ingredients of the safety data sheet were included in the risk assessment in order to create a worst case scenario.

The risk assessments were conducted under the following assumptions: a ready-to-use spray product is used at dental workplaces and creates a visible spray haze. The spray is used between one and 30 min per day and at 2–3 days per week. It is used in the breathing zone of an employee and in a room of less than 100 m^3^, which has no general ventilation. No protective measures, other than a normal breathing mask and gloves are used. The results are represented according to the singular ingredients of the dental sprays excerpted from the safety data sheet.

The results are reported in a table with the following column-entries:

- hc (hazard class): different types of nano-particles may cause different health effects. Therefore, the different particles are classified into hazard classes (hc), based on known information about hazardous properties. Class A includes the least hazardous substances, class E is the most hazardous.
- ec time (exposure class time): exposure class is estimated by the model using information on amount of product being released, type of operation, distance to the source and taking the duration and frequency of the task into account. Class 1 represents the lowest exposure, class 4 the highest.
- risk time (and frequency-weighted): indicates the health risk taking duration and frequency of exposure into account. Stoffenmanager only deals with risks of dangerous substances. These risks are assessed by the hazard of the substance and the exposure to the substance. Risk score III indicates a low risk, risk score I a high risk. First the hazard class is determined, then exposure during the task is assessed. Finally a risk score is calculated based on these assessments.
- ec task (exposure class task): Exposure class (ec) is estimated by using information on amount of product release during the task, type of operation and distance to the source. Class 1 stands for lowest exposure, class 4 for highest exposure.
- risk task (−weighted): indicates the health risk taking duration and frequency of exposure into account. Stoffenmanager only deals with risks of dangerous substances. These risks are assessed by the hazard of the substance and the exposure to the substance. Risk score III indicates a low risk, risk score I a high risk. First the hazard class is determined, then exposure during the task is assessed. Finally a risk score is calculated based on these assessments.

### Experimental setting (particle generation, sampling, and analysis)

**Fig. 1:**
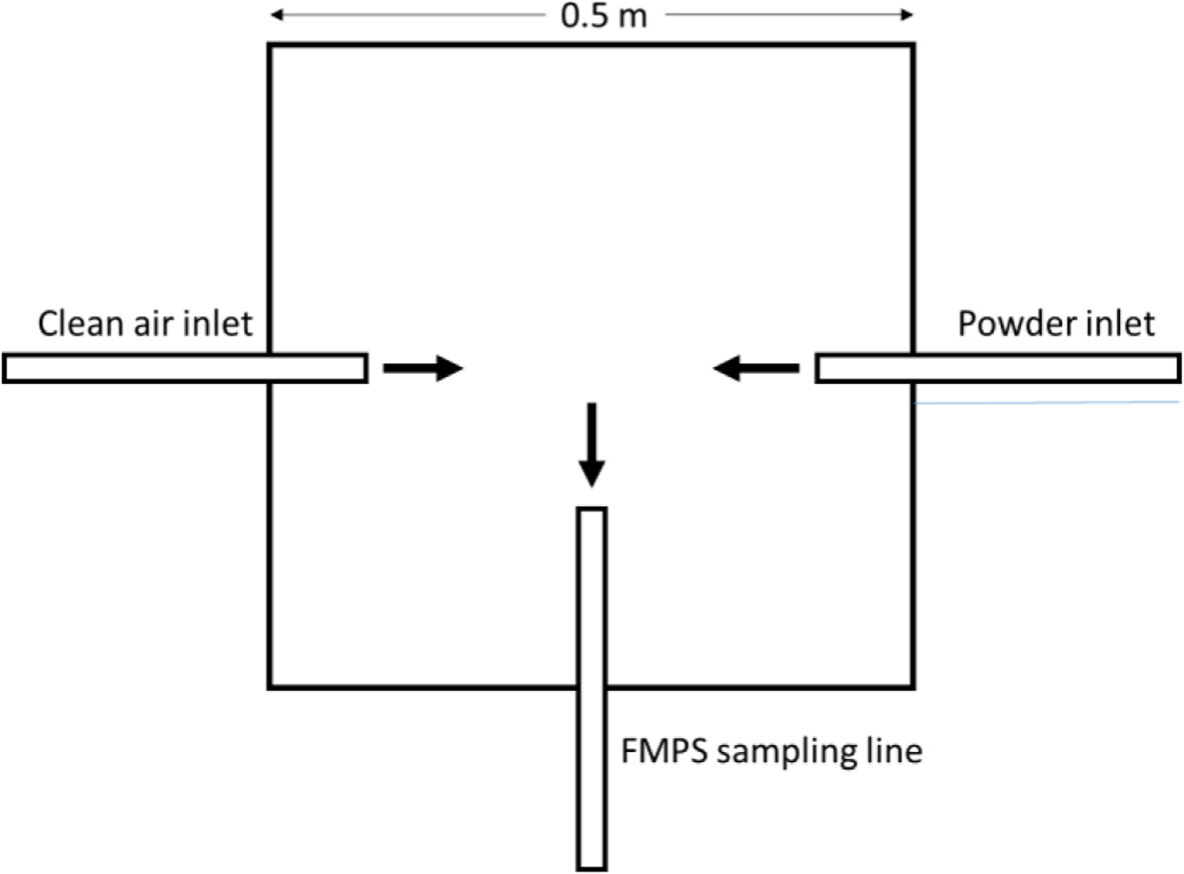
Top view of the mixing chamber (aluminum cube with a side length of 0.5 m)

To examine the particle size distributions of the sprays under standardized conditions, an examination protocol was developed based on other experiments with welding aerosols [24]. The powder-nebulizers of the spray systems were connected to an aluminum mixing chamber (0.5 m × 0.5 m × 0.5 m) which was flushed with about 20 L/min filtered air. The dimensions of the mixing chamber were chosen to simulate the breathing zone of dental personnel, who usually work in a forward flexion of trunk and head [25]. From this chamber the FMPS – air sample (10 L/min) was taken. Figure 1 shows a top view of the mixing chamber. Once a stable background concentration was established for about 5 min, the aerosol was introduced by conducting one spray application procedure. Each nebulizer was activated for 3 s and then particle size distributions were measured for about 5 min and 1 min averages were calculated.

### Particle size analysis

In the experimental setup, we assessed particle number-size distributions and total number concentrations using a fast mobility particle sizer (FMPS—model 3091, TSI, USA) was used [26, 27]. This device classifies different particle sizes (from 6 nm to 600 nm) simultaneously according to their electrical mobility. The possibility of simultaneous measurements in 32-size channels with a time resolution of 1 s of this device makes it suitable for the measurement of fast changing, unstable aerosols.

### Data analysis

To compare particle size distributions, the second one-minute-average-distribution was used. The first minute was rejected in order to be sure that propellant droplets, which were produced during nebulization, were completely evaporated. Each new measurement started after particle size concentration in the chamber returned to the background level of the starting point. In most cases we waited for approximately 5 min for the return to the background level of approximately 1000 particles/cm^3^. This value is comparable to other experimental measurements for dentists [9]. For each one-minute-average-distribution the total particle number concentration (C_tot_) and the fraction of particle number of particles smaller than 100 nm (*F*_< 100_) was calculated. Measurements were repeated four times for each nebulizer.

### Modelling particle deposition in the human respiratory system

A mathematical model used to estimate the total and regional lung deposition of particles is the MPPD model (multiple-path particle dosimetry) ([28], ARA). In this study, we used the MPPD model (version 3.04) to roughly estimate the deposition fraction of particles by number in the extra-thoracic region, the trachea-bronchial region, and the alveolar region, and the entire lung for adults. The model was used for particle size diameters between 6 up to 600 nm, as this was the range of the recordings of our measurements. As model entry parameters we refer to the work of Vu et al. [29] and use the reference respiratory values for light exercise for Caucasian people, as we assume that it best fits the working conditions in a dental practice. Mean particle sizes of the different sprays were entered in the model. Wherever input parameters were not experimentally available, the default values in the MPPD software were used.

## Results

### Information gathered from safety data sheets

General information collected from safety data sheets on ingredients, exposure controls, and application procedures of the different scanning sprays are presented in Table 1. These data – along with the reported mass fraction (if it was reported) – were later on included in the risk assessment tool “Stoffenmanager.Nano”.

### Results of the experimental measurements

**Fig. 2:**
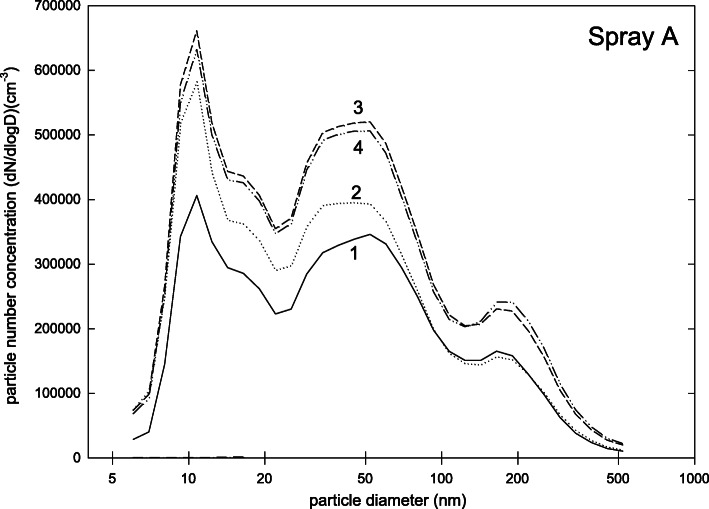
Particle size distribution (dN/dlogd) during measurements for product A (four repetitions of measurement)

The particle size distribution for spray A is shown in Fig. 2. For this product a reproducible three-modal distribution was found with one mode at about 10 nm, one mode at about 50 nm, and one mode at 200 nm. Total number concentration was on average (mean ± standard deviation) (5.3 ± 1)·10^5^ cm^− 3^. On average 83% of the detected particles were smaller than 100 nm (Table 2). The safety data sheet of spray A did not contain information on particulate matter or pigments. As a potentially hazardous substance, pentane was denoted.

**Table 2:** Mean and standard deviation (StD) of the fraction of particles smaller than 100 nm (*F*_< 100_) and total number concentration for the dental sprays under investigation

Sprays	*F*_< 100_		Total number concentration
	Mean (%)	StD (%)	
Mean background concentration			(780 ± 580) cm^−3^
Spray A	83.0	1.6	(5.3 ± 1.0) * 10^5^ cm^− 3^
Spray B	73.2	1.1	(3.3 ± 0.4) * 10^5^ cm^− 3^
Spray C	93.1	2.8	(5.0 ± 0.5) * 10^5^ cm^− 3^
Spray D	15.6	15.0	(1.3 ± 0.5) * 10^5^ cm^− 3^
- Propellant D alone	100.0	0.0	(0.3 ± 0.2) * 10^5^ cm^− 3^
Spray E	9.0	6.9	(2500 ± 900) cm^− 3^

**Fig. 3:**
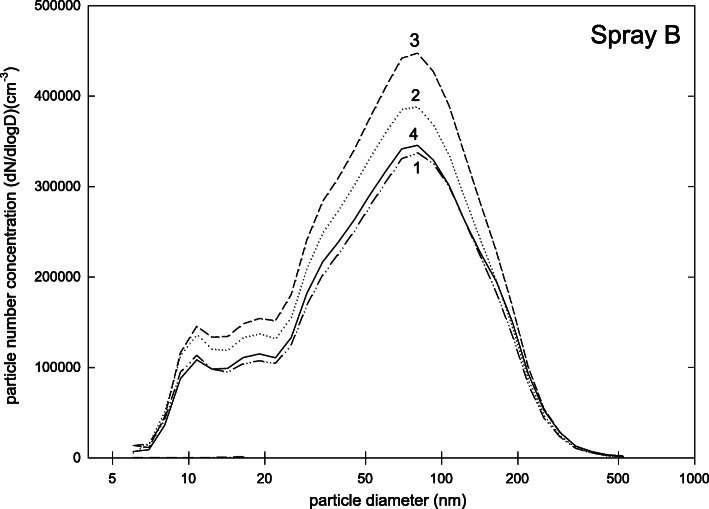
Particle size distribution (dN/dlogd) during measurements for spray B (four repetitions of measurement)

For spray B (Fig. 3) fewer particles were found in the size range below 20 nm. The size distribution was dominated by a distinct peak at about 100 nm. A considerably smaller peak was found at about 10 nm. The fraction of particles below 100 nm was 73% and the total number concentration (3.3 ± 0.4)·10^5^ cm^− 3^. The safety data sheet stated talcum as particulate ingredient. It also mentioned citrus oil and naphta as ingredients. Titanium dioxide, silicium dioxide, talcum and ethanol were denoted as ingredients with a need for exposure control.

**Fig. 4:**
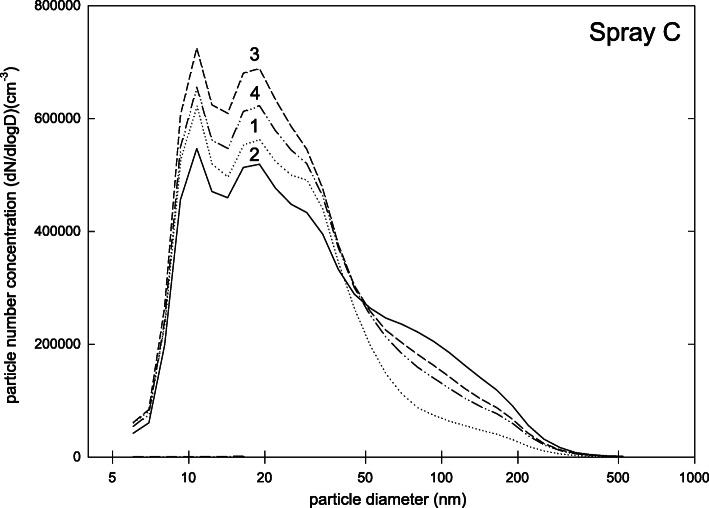
Particle size distribution (dN/dlogd) during measurements for spray C (four repetitions of measurement)

Particles emitted by spray C were considerably smaller (Fig. 4) than those of sprays A and B with two peaks at about 10 and 20 nm. Total number concentration was (5.0 ± 0.5)·10^5^ cm^− 3^ and the fraction of particles smaller than 100 nm was 93%. The safety data sheet reported pentane with non-hazardous additions as ingredients.

**Fig. 5:**
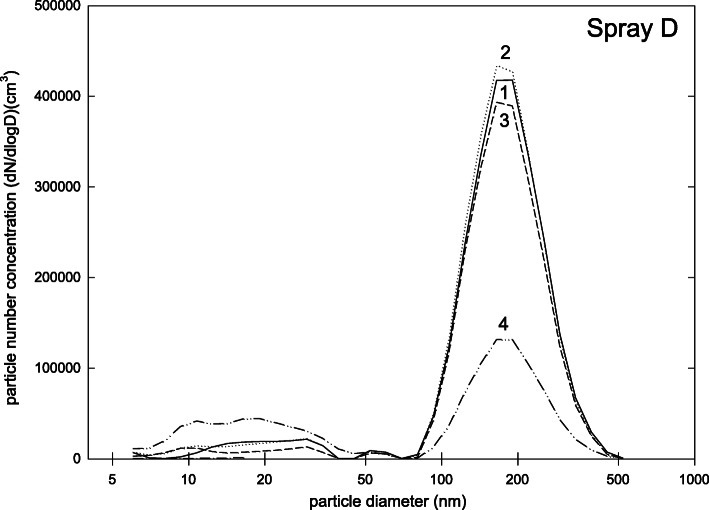
Particle size distribution (dN/dlogd) during measurements for spray D (four repetitions of measurements)

**Fig. 6:**
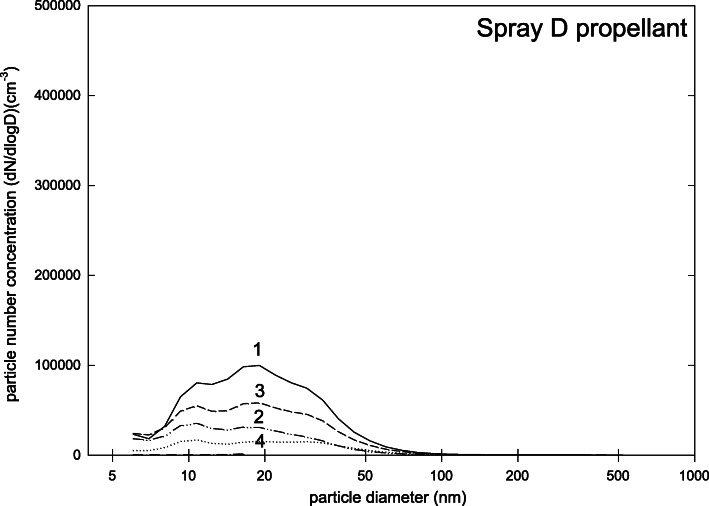
Particle size distribution (dN/dlogd) during measurements for propellant of spray D (four repetitions of measurement)

Spray D has a different operating principle than the devices mentioned before. While the aforementioned sprays are for sale as mixture of propellant and other ingredients in one container, in spray D powder and propellant are stored in different containers, and are only mixed during the spraying process. This leads to a different particle size distribution (Fig. 5). The main peak of the size distribution is at about 200 nm whereas a broad distribution was found between 6 and 50 nm. Only 16% of the particles were smaller than 100 nm, though, and the total number concentration was only (1.3 ± 0.5)·10^5^ cm^− 3^. The safety data sheet listed titanium dioxide and zirconium oxide, as well as talcum as ingredients and substances with a need for exposure control. Since the propellant for spray D (which consists only of isobutene) is bottled in a separate container it was possible to measure the size distribution of propellant particles. This size distribution is shown in Fig. 6. Again, a broad distribution between 6 and 50 nm was found, indicating that the similar particle fraction found in Fig. 5 was due to propellant, while the 200 nm peak seems to belong to the particulate ingredients. All propellant particles were smaller than 100 nm but the total number concentration was only (0.3 ± 0.2)·10^5^ cm^− 3^. The respective safety data sheet reports isobutene as ingredient and substance with need for exposure control.

**Fig. 7:**
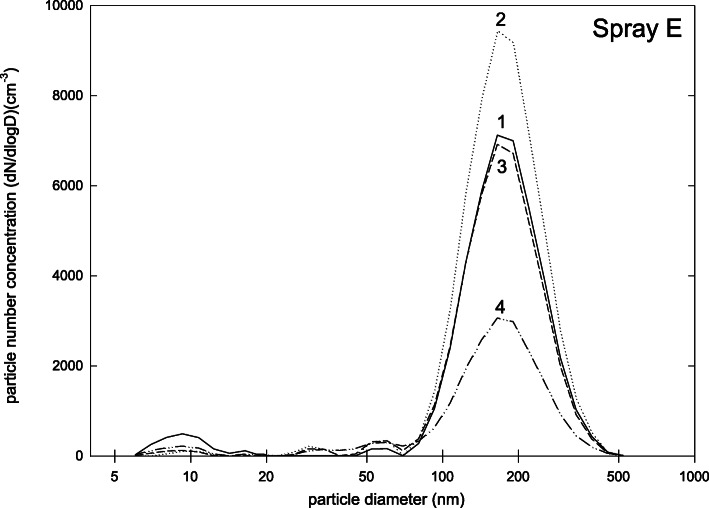
Particle size distribution (dN/dlogd) during measurements for Product E (four repetitions of measurement)

Finally, spray E uses yet another operating principle, where particles are propelled into the oral cavity by an electrically driven pressured air source. Again the main particle peak was at about 200 nm. Only 9% of the particles were smaller than 100 nm and the total number concentration was with (2500 ± 900) cm-3 very low (Fig. 7). The safety data sheet of this spray lists titanium dioxide, zirconium dioxide and zinc distereate as ingredients and titanium dioxide as substance with exposure control (Table 1).

Table 2 summarizes the detailed findings of the standardized measurements.

### Results from risk assessment with “Stoffenmanager.Nano”

From a practical point of view, the use of intraoral scanning sprays should be accompanied by the performance of a workplace risk assessment. As our experimental measurements indicated that the intraoral spray applications can lead to inhalation exposure to ultrafine particles, we used the “Stoffenmanager.Nano” tool for a rough estimate of the risks related to the use of the matting sprays. The results indicate that at a hypothetical dentist’s workplace, the use of intraoral matting sprays can pose a high health risk, depending on the ingredients used in the sprays and the preventive measures taken. It should be mentioned in this context that Stoffenmanager per se only deals with risks of dangerous substances, as categorized by the safety data sheets or other sources. We refer to categorization by safety data sheets and MAK-values (maximale Arbeitsplatzkonzentration, DFG). The final “Nanomanager” risk score (I – III) is calculated based on the hazard of the substance and the exposure to the substance, and while a risk score III indicates a low risk, a risk score I indicates a high risk.

With regard to hazard of the substance, titanium dioxide, zirconium dioxide, silicium dioxide, and talc pose the greatest health risks as they are considered to have carcinogenic potential (hazard class D). While silicium dioxide is graded as a class 1 carcinogen, talc belongs to carcinogen class 3B and titanium dioxide and zirconium dioxide were graded as carcinogens class 4 which means that compliance to MAK-values [30] reduces the probability for carcinogenic risk. Carcinogenic risk of titanium and zirconium dioxide has to be regarded differently, if particles sizes are below 100 nm. Other substances are harmful or irritating (B) or classified as toxic, corrosive and/or inhalation allergen (C).

Please note that the risk assessment of Table 3 refers to a worst case scenario, as we hypothesized that no preventive measures (except wearing a breathing mask) were installed. But this seems to be a realistic scenario in dental practice. With this in mind, the use of spray D and E led to a task-weighted risk assessment of I, which represents the highest risk group and indicates an urgent need for preventive measures. Apart from that, sprays with organic ingredients (sprays A and C) are categorized in risk group III, whereas spray B is categorized in risk group II.

**Table 3:** Results of risk assessment with “Stoffenmanager.Nano”

Product	Nano component	hc	ec time	Risk time	ec task	Risk task
Spray A	pentane	B	1	III	1	III
Spray B	talc	B	2	III	2	III
	naphta	D	2	II	2	II
	lemon oil	C	1	III	2	II
Spray C	pentane	B	1	III	1	III
Spray D	titanium dioxide	D	2	II	3	I
	zirconium dioxide	D	2	II	3	I
	zinc distereate	C	2	II	2	II
	isobutane (propellant)	A	2	III	3	III
Spray E	titanium dioxide	D	2	II	3	I
	zirconium dioxide	D	2	II	3	I
	zinc distereate	C	2	II	2	II
	silicium dioxide	D	1	II	2	II

*hc* Hazard class: class A -least hazardous to class E - most hazardous substances

*ec* Time (exposure class time): class 1 - lowest exposure, class 4 – highest exposure

risk time: risk score III - low risk, risk score I - high risk. ec task (exposure class task): class 1 - lowest exposure, class 4 - highest exposure

risk task (−weighted): risk score III - low risk, risk score I - high risk. (for further explanation, please see Methods section)

### Deposition model

**Fig. 8:**
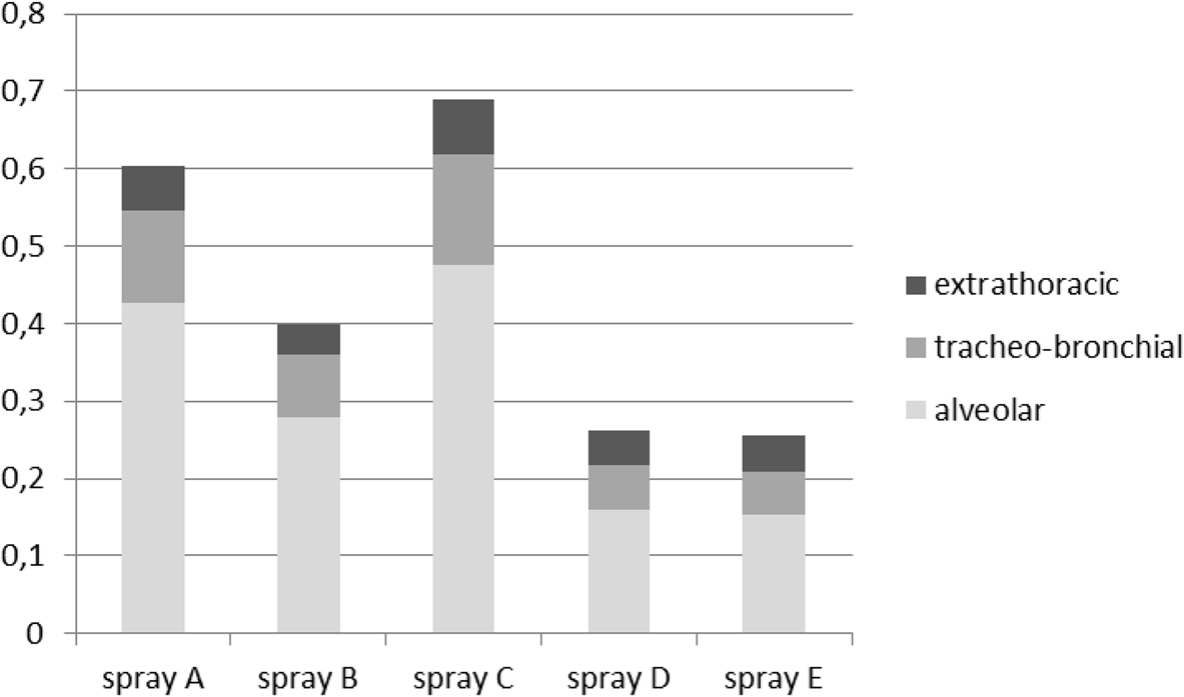
Deposition fraction of particle number in regions of the adult lung

Total and regional lung deposition fractions for the examined matting sprays for adults with light physical activity (at the workplace) are shown in Fig. 8. The total lung deposition fraction ranged between 0.256 (spray E) and 0.689 (spray C). Matting sprays with powder ingredients (sprays B, D, and E) show a mean size range between 100 to 200 nm. They are less likely to lead to particle deposition in the alveolar region compared to particle sizes below 100 nm. In combination with exposure assessment it becomes obvious that the sprays containing particles with higher hazard classes are less likely to reach the alveolar region (deposition in alveolar region for different sprays: 0.426 (spray A), 0.279 (spray B), 0.477 (spray C), 0.159 (spray D), 0.153 (spray E)).

## Discussion

In dentistry, spray applications are used to coat dental surfaces with anti-reflecting substances, e. g. to take optical impressions for dental restorations. These spray applications lead to inhalation exposures to fine and ultrafine particles of dentists and patients, and inhalation of these particles is assumed to be associated with detrimental health consequences for humans [31].

Until now, too little is known about exposures associated with the use of dental matting sprays [9], to conduct meaningful risk assessments and finally decide on preventive workplace measures. This study tries to fill in some of the gaps of knowledge for exposure or risk assessments at dental workplaces. To get a closer look at the potential exposures of patients and health care personnel, we measured particle size distributions and number concentrations of different dental matting sprays used in everyday practice. We also conducted a risk assessment for a hypothetical dentists’ workplace using the information of safety data sheets and other sources for judging hazardous substances. Finally, we used the results of our measurements for a crude estimation of particle deposition in the extra-thoracic, thoracic, and alveolar region after inhalation exposure to the examined dental matting sprays.

In general, our experiments focus on measuring particle sizes and area properties of fine and ultrafine particles. Especially ultrafine particles may have different sizes, shapes, chemistry, and crystalline structure than their bigger sized counterparts [32]. Though more recent articles have shown that surface area alone may not dictate toxicity [33–37], our measurement device dictated a focus was on particle size and surface area. We are aware, that e. g. deposition modelling would have improved with more detailed information on other parameters, e. g., effective density or Geometric Standard deviations.

In order to create reproducible measurements of particle size distributions and number concentrations, we were using a flushed mixing chamber and calculated mean values for comparison. The protocol of the methodic approach based on other research of fine and ultrafine welding fumes [24] and was designed to simulate the breathing zone of a dentist. Though this experimental setup created reproducible results with regard to size distributions, the number concentration varied greatly. As we controlled the background values in the mixing chamber, we assume that the non-time-dependent differences in particle number are based on a random distribution of the spray mist in the flushed mixing chamber. Note that the results of the underlying experiment as well as those of other authors diverge in approximately the same magnitude [24, 38]. Another negative aspect of the experimental setup is that it merely results in relative values, which cannot be extrapolated to absolute numbers of particles.

Nevertheless, our results demonstrate that dental matting sprays can release fine and ultrafine particle fractions to be inhaled at dental workplaces. This result supports the earlier experiments of Rupf et al. [9] at dental workplaces, but also that of other authors who examined consumer-based spray applications [10]. But the patterns of size distributions and number concentrations should be given some thought: While a relevant fraction of particles of sprays A and C was below 100 nm of size (83 and 93%, respectively), this result was not true for sprays D and E (16 and 9% respectively). It can be argued that the reasons for this difference are the different forms of application. As sprays A and C only report organic ingredients, while sprays D and E report mainly particles like titanium and zirconium dioxide, these differences can also occur due to different ingredients of the aerosols. The size pattern of spray B is somewhere between the above mentioned two groups as it shows a distinct peak but at smaller sizes than sprays D and E.

Mathematical modelling of exposures showed that lung deposition ranged between 26 and 69%. Vu et al. [29] reported lung deposition fractions of indoor activities like vacuum cleaning, which were even higher (up to 73%). The modelling results therefore seem to be realistic. Note that the smaller fractions are associated with bigger particle sizes, while smaller particle sizes are associated with higher deposition rates. According to the risk assessment the sprays with the smaller size distributions, which can present propellants’ residua, are less hazardous than those with the bigger size distributions.

Propellants, which are often included a-priori in a ready-to-use spray mixture, may pose a relevant source of the ultrafine fraction of our measurements. This assumption was supported by comparing particle size distributions of spray D in general with the particle size distribution of the propellant of spray D (Figs. 5 and 6). Here, we found that the pattern of particles with diameters < 100 nm was more or less identical to the pattern seen when analyzing the propellant only. Therefore we concluded that – despite the relevant airflow in our mixing chamber and a “drying time” of 1 min – the ultrafine fraction in the mixing chamber was related to propellants. Note that other authors also report effects of vapour molecules or atom clusters of test aerosols [39]. One explanation for this artefact can be that propellant particles are gathered on the filters of the FMSP but later evaporate. This discrepancy would overestimate the overall number concentration of nanoparticles as well as shift the particle size distribution to the lower size range. The degree of discrepancy may depend on the properties of vapour constituents [39].

With our experimental setup, contents of the ultrafine fractions, i. e. the fractions below 100 nm, are not entirely clear. They could include pigments e. g. titanium dioxide particles, as well as talc or propellant. Therefore, our results can only point out a general occupational health risk when using matting sprays, considering the results of the risk assessments with “Stoffenmanager.Nano”. Some of the matting sprays contain ingredients like titanium and zirconium dioxide and silicia dioxide, which have been identified as health hazards for the lung, especially in an ultrafine form. In nanoparticle research, especially titanium dioxide is under scrutiny. Though there is currently no epidemiologic proof for a dose-response relation between ultrafine TiO_2_ and lung cancer in TiO_2_-exposed workers, animal studies (rats) have shown dose-response relationships for ultrafine TiO_2_ and cancerous, as well as inflammatory endpoints [40–42]. Therefore, more information about ultrafine toxicity and effective preventive means are needed to reduce health threats at the workplace.

While our risk assessments rely on the information of safety data sheets, we are aware that transmission electron microscopy (TEM, [43]) would have supported the characterization of ingredients for risk assessments. But TEM results will hardly be available for risk assessments in real life, therefore, we decided to only rely on the information available in practice, and supplement them with standardized examinations.

All in all, the increasing use of nanomaterials in consumer and medical products warrants in depth research of according health risk [10]. Currently, risk assessments are difficult, as information on safety data sheets is insufficient for risk assessment of ultrafine ingredients. Measurements of ultrafine fractions are still technically challenging and need expensive equipment. Only little is known about the ingredients measured in the ultrafine fractions. More sophisticated approaches and the use of elaborate measurement techniques may help in gaining a better understand. Nevertheless, practical risk assessments call for other approaches.

## Conclusion

Some dental powders contain relevant fractions of ultrafine particles of unclear content, which might pose a health risk for dental personnel. Because of generalized information in safety data sheets (e. g. “pigment suspension”), the exact contents of dental sprays, and, what is even more important, the exact content of the ultrafine fraction cannot yet be accounted for. Particle size analysis can bring out comparable patterns and help creating a better understanding of associations between ingredients and size patterns. In our experiments e. g., particulate matter was associated with bigger sized particles compared to sprays with a huge portion of propellant as “ingredient”. Therefore, and because of the influence of propellants might be interfering with the results, the ingredients of the nanoscale fraction are not clear. Detrimental effects on human health by dental sprays can therefore not be ruled out. Other authors [9] reported relevant exposure to fine and ultrafine particles for patients and dental personnel during the use of dental sprays, nevertheless, the size distribution and particle content of the ultrafine fraction is also not entirely clear in their work. Therefore, additional analysis, e. g. with transmission electron microscopy will be needed to amend this situation and to improve patient and occupational safety. Furthermore, ideas for practical risk assessments are needed.

## Data Availability

The datasets of the current study are available from the corresponding author on reasonable request.
